# Lessons learned and questions raised during and post-COVID-19 anthropopause period in relation to the environment and climate

**DOI:** 10.1007/s10668-020-01075-4

**Published:** 2020-11-19

**Authors:** Christos S. Zerefos, Stavros Solomos, John Kapsomenakis, Anastasia Poupkou, Lida Dimitriadou, Iliana D. Polychroni, Pavlos Kalabokas, Constandinos M. Philandras, Dimitris Thanos

**Affiliations:** 1 Research Centre for Atmospheric Physics and Climatology, Academy of Athens, Athens, Greece; 2 Biomedical Research Foundation, Academy of Athens, Athens, Greece; 3 Navarino Environmental Observatory (N.E.O.), Messinia, Greece; 4 Mariolopoulos-Kanaginis Foundation for the Environmental Sciences, Athens, Greece; 5 Faculty of Geology and Geoenvironment, National and Kapodistrian University of Athens, Athens, Greece

**Keywords:** COVID-19, Pandemics, Climate change, Air quality, Anthropopause

## Abstract

In the first part, this work reports that during the global “anthropopause” period, that was imposed in March and April 2020 for limiting the spread of COVID-19, the concentrations of basic air pollutants over Europe were reduced by up to 70%. During May and June, the gradual lift of the stringent measures resulted in the recovery of these reductions with pollution concentrations approaching the levels before the lockdown by the end of June 2020. In the second part, this work examines the alleged correlations between the reported cases of COVID-19 and temperature, humidity and particulate matter for March and April 2020 in Europe. It was found that decreasing temperatures and relative humidity with increasing concentrations of particulate matter are correlated with an increase in the number of reported cases during these 2 months. However, when these calculations were repeated for May and June, we found a remarkable drop in the significance of the correlations which leads us to question the generally accepted inverse relation between pandemics and air temperature at least during the warmer months. Such a relationship could not be supported in our study for SARS-CoV-2 virus and the question remains open. In the third and last part of this work, we examine the question referring to the origin of pandemics. In this context we have examined the hypothesis that the observed climate warming in Siberia and the Arctic and the thawing of permafrost could result to the release of trapped in the permafrost pathogens in the atmosphere. We find that although such relations cannot be directly justified, they present a possible horrifying mechanism for the origin of viruses in the future during the developing global warming of our planet in the decades to come. Overall the findings of our study indicate that: (1) the reduction of anthropogenic emissions in Europe during the “anthropopause” period of March and April 2020 was significant, but when the lockdown measures were raised the concentrations of atmospheric pollutants quickly recovered to pre-pandemic levels and therefore any possible climatic feedbacks were negligible; (2) no robust relationship between atmospheric parameters and the spread of COVID-19 cases can be justified in the warmer part of the year and (3) more research needs to be done regarding the possible links between climate change and the release of new pathogens from thawing of permafrost areas.

## Introduction

The recent unprecedented pandemic crisis is so large, that the scientific community has been tempted to investigate the possible links and feedbacks between COVID-19 and the environment (Gautam and Hens 2020). Obviously, the pandemic has many negative effects influencing our lives, health and the economy (Wang et al. 2020). However, COVID-19 had also one positive effect for the atmospheric environment, namely the reduction of air pollution at several parts of the world due to the lockdown measures (Liu et al. 2020a, b; Muhammad et al. 2020). Paradoxically, this improvement in air quality may be beneficiary for the health and well-being of the local populations despite the pandemic risk. On the other hand, most of the reduction in anthropogenic emissions was associated with the transport sector, while industrial pollution sources and adverse weather conditions were still found to increase the concentrations of air pollutants at specific areas (e.g., Wang et al. 2020). Significant improvements in air-quality conditions have already been reported for India (Gautam et al. 2020; Gautam 2020a, b), China (Dutheil et al. 2020; Gupta et al. 2020) and for specific cities like Delhi, London, Paris and Wuhan (Bherwani et al. 2020).

In this study, we focus on the links and feedbacks between COVID-19 and the environment both during and after the lockdown period based on station measurements over Europe. In this manner, we examine both the effects of the COVID-19 lockdown measures on the atmospheric environment and vice versa, i.e., the possible effects of atmospheric variables on pandemic spread. We present air quality measurements of NO_2_, CO, O_3_, and PM2.5 concentrations in Europe during the lockdown phase (March–April 2020) and during the recovery phase (May–June 2020). To the best of our knowledge, this is the first study to report the variability in air-quality conditions, before, during, and after the lockdown period, based on station measurements over the entire Europe. This “anthropopause” period imposed by COVID-19 measures provided a formidable opportunity to study air pollutants in Europe under the conditions of reduced anthropogenic activity. Air quality measurements are discussed in the first part of this work where we found out that air pollutants have been reduced at levels normally anticipated not before 2050 if the European Union Green Agenda is fully implemented. Next this work tackles important questions related to the alleged interrelations between environmental conditions (temperature, moisture, PM2.5 concentrations) and the spread of COVID-19 cases. The last part of the paper examines a hypothesis, according to which the observed climate warming at high latitudes and the thawing of permafrost could facilitate the release of pathogens in the atmosphere.

## Air quality during the anthropopause period in Europe

**Fig. 1:**
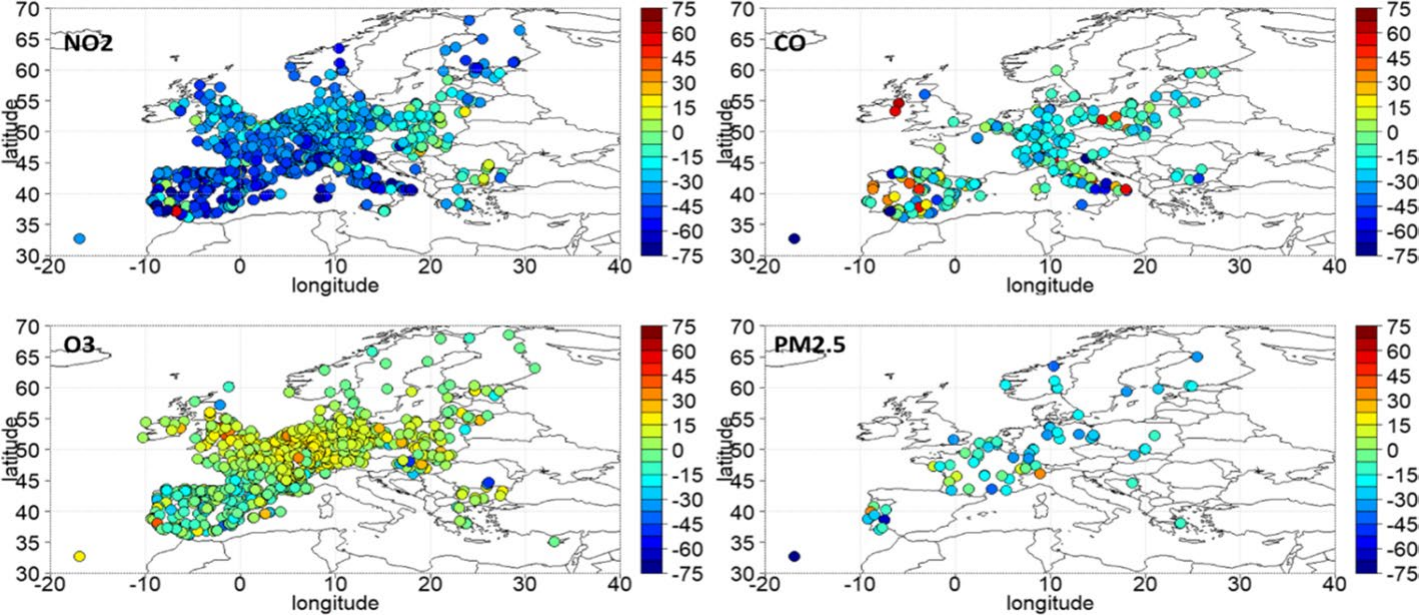
March–April 2020 percentage anomalies from 2015 to 2019 climatology based on Eionet network

**Fig. 2:**
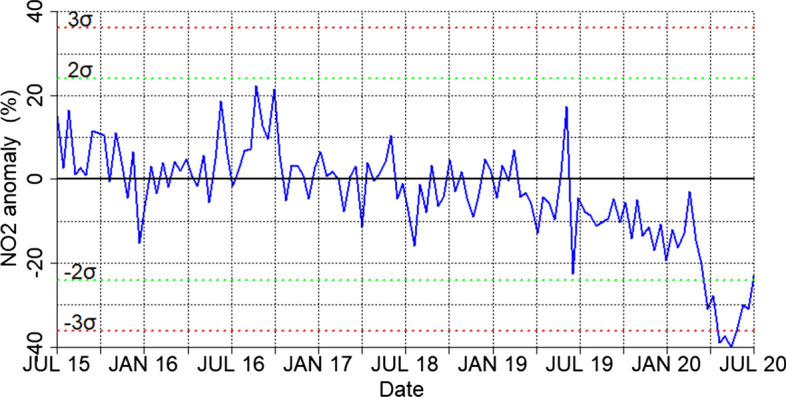
Time series of percent anomalies for biweekly mean NO_2_ concentrations in Europe during the period 2015–2020, based on the Eionet network

A global anthropopause period was imposed through the lockdown due to the COVID-19 pandemic outbreak. During March–April 2020, the lockdown of transport and various businesses related activities have resulted in significant reduction of emissions. We have used the Eionet platform (https://discomap.eea.europa.eu/map/fme/AirQualityExport.htm) air quality data provided by the European Environmental Agency (EEA), to study the anomalies (in percentage) of 4 selected air pollutants. Τhe percentage anomalies for NO2, CO, O3 and PM2.5 are shown in Fig. 1. In this figure, the anomalies were calculated in percent from the mean of the 5-year period 2015–2019. As can be seen from Fig. 1, during the lockdown of transport and other activities the concentrations of basic air pollutants such as NO2, CO, PM2.5 have all dropped in the range from − 10 up to − 60 or − 70%. As expected, surface O3 levels have increased by more than 10% in most parts of Europe, which is observed in the most polluted regions of the continent and at urban stations due to the reduction in the main ozone destruction mechanism in urban environments (NO titration) following the substantial decrease of NO_x_ emissions (Finlayson-Pitts and Pitts 1997). These changes were unprecedented and so coherent spatially, that the Europe as a whole had air quality characteristics foreseen to occur only after the possible implementation of European decisions that will take place until 2050 towards a neutral carbon environment. Figure 2 shows the time series of percent anomalies for NO_2_ during the period June 2015–June 2020, based on the Eionet network. The points in the timeseries represent biweekly NO_2_ concentration percentage departures from the corresponding 5-year (2015–2019) mean during the period 2015–2020. The horizontal dashed lines indicate ± 2*σ* and 3σ confidence levels. From that figure one can easily see the overall European response of NO_2_ concentration on a biweekly basis from July 2015 to July 2020. The abrupt and highly significant drop in NO_2_ concentration is evident during the antropopause period (March and April 2020) followed by a recovery phase in May and June 2020. It is important to mention here that the observed changes to the concentrations of the above short-lived pollutants are mostly relative to local air-quality considerations. Any possible impacts on the actual global climate would require much longer periods of emission limitations before any expected effect can be detected.

## The large-scale spread of infections and the alleged role of atmospheric parameters in Europe

The COVID-19 outbreak has caught by surprise China’s Wuhan area before Christmas 2019 where stringent measures forced people to stay at home. The population in the Western world started being affected much later close to February 2020. Stringent measures were not taken worldwide and in fact WHO has characterized COVID-19 as pandemic on 12 March 2020 (World Health Organization—WHO 2020). In Europe the first cases followed a day to day increase in geometrical progression throughout the month of March 2020 which also marks the beginning of the declining phase in China. Some countries took early measures (such as Greece), while others implemented measures later on. Some took no measures at all, such as Sweden. A detailed table which includes the measures taken by different countries is provided in Appendix Table 4.

Here we examine the alleged interrelationship between atmospheric temperature, humidity, particulate matter concentrations and the mean daily number of COVID-19 cases at individual European countries and in Europe as a total. Although the global spread of the infection occurs primarily through social contact, traveling, aviation, trade, etc., the variability of meteorological conditions could also be related to the changes in the daily reported number of cases. The role of environmental conditions on infection spread has been previously reported for pandemics ( Hemmes et al. 1960; Kissler et al. 2020; Lin et al. 2006). Recent publications address the relation of COVID-19 cases with the meteorological conditions either on a global scale (Huang et al. 2020; Lal et al. 2020; Sobral et al. 2020) or at specific cities and countries Benedetti et al. 2020; Briz-Redón and Serrano-Aroca 2020; Demongeot et al. 2020; Gunthe et al. 2020; Menebo 2020; Şahin 2020). Other papers have studied separately the effects of meteorology from those of air pollution on the number of COVID-19 infections or deaths in Europe (Ogen 2020; Zoran et al. 2020a, b) and elsewhere (Wang et al. 2020).

In this context, we have re-examined the relationship that was alleged in some of the above papers to exist between air temperature, humidity and daily number of new COVID-19 cases. In that sense, we have correlated the day-to-day variability of COVID-19 cases with major environmental parameters such as the daily mean temperature and relative humidity and the daily mean concentrations of suspended particulates with diameter less than 2.5 μm. Meteorological and air quality data for the regression analysis were taken from Copernicus ERA5 reanalysis (Hersbach et al. 2020) and CAMS NRT Global, respectively (https://apps.ecmwf.int/datasets/data/cams-nrealtime/levtype=sfc/). The sources for pandemic COVID-19 cases are shown in Appendix Table 5 (last update July 2020). In view of the difficulties involved in reporting the cases on time for every day, we decided to calculate the 5-day average for each dataset which pertains to a given region and country in Europe. The number of regions for each country are shown in Tables 1 and 2 which also show the number of pairs entering each correlation. For each 5-day period, we calculated the average number of new reported COVID-19 cases normalized by the population of each region which were afterwards correlated with the climatic anomalies of the atmospheric parameters (T, RH) together with PM2.5 daily mean concentrations. The correlations were calculated for 18 European countries as shown in both Tables 1 and 2, where the Balkans include Slovenia, Croatia, Bosnia-Herzegovina, Montenegro, Albania and Greece. We should note here that different countries have different testing rates. Low testing rates may well mask the true infection numbers. However the large number of pairs entering our correlations (in Tables 1 and 2) ensures that statistically speaking, the significance of the correlations ensures the percentage of variance explained by environmental parameters without refering to the true infection numbers. The last column in Tables 1 and 2 shows the correlations for Europe as a whole between COVID-19 cases, temperature, relative humidity, PM2.5. More specifically, Table 1 shows the above-mentioned correlations for individual months (March, April, May, June), while Table 2 shows the correlations for the 2 bimonthly samples (March–April and May–June) as well as the 4-month period (March–June 2020). Statistically significant correlations at better than the 99% confidence level are shown by an asterisk next to the correlation coefficent. Both Tables 1 and 2 show in parentheses for each country and for the Europe as a whole, the number of regions from which data were provided and in parenthesis the number of pairs entering each correlation.

**Table 1:** Correlation coefficients between the number of new COVID-19 cases during a 5-day period and the mean 5 days temperature, relative humidity, PM2.5 and CVI for each month from March to June 2020

	Portugal (7 regions)	Spain (19 regions)	Italy (21 regions)	U.K. (10 regions)	France (13 regions)	Belgium (3 regions)	Netherlands (12 regions)	Germany (15 regions)	Swiss (23 regions)	Poland (15 regions)	Sweden (21 regions)	Norway (11 regions)	Balcans (6 regions)	Europe (176 regions)
*March*														
Temperature	− 0.18 (35)	− 0.33* (105)	− 0.45* (95)	− 0.07 (50)	− 0.38* (65)	− 0.7* (15)	− 0.47* (60)	− 0.3* (80)	− 0.12 (115)	− 0.22 (80)	0.02 (105)	0.03 (55)	0.29 (28)	− 0.14* (860)
Relative humidity	0.02 (35)	− 0.03 (105)	0.07 (95)	− 0.38* (50)	− 0.59* (65)	− 0.9* (15)	− 0.68* (60)	− 0.64* (80)	− 0.34* (115)	− 0.7* (80)	− 0.47* (105)	0.12 (55)	− 0.08 (28)	− 0.12 (860)
PM 2.5	0.36 (35)	0.17 (105)	0.2 (95)	0.47* (50)	0.29 (65)	0.5 (15)	0.47* (60)	0.28 (80)	0.38* (115)	0.37* (80)	0.07 (105)	− 0.11 (55)	0.72* (28)	0.22* (860)
CVI	0.36 (35)	0.37* (105)	0.43* (95)	0.43* (50)	0.46* (65)	0.87* (15)	0.69* (60)	0.52* (80)	0.43* (115)	0.6* (80)	0.21 (105)	− 0.16 (55)	0.65* (28)	0.29* (860)
*April*														
Temperature	− 0.23 (42)	− 0.47* (126)	− 0.68* (114)	0.06 (60)	− 0.46* (78)	− 0.16 (18)	0.14 (72)	− 0.19 (96)	− 0.39* (138)	0.1 (96)	0.2 (126)	− 0.26 (66)	0.05 (36)	− 0.17* (1032)
Relative humidity	0.27 (42)	− 0.4* (126)	− 0.22 (114)	0.28 (60)	− 0.51* (78)	− 0.29 (18)	− 0.42* (72)	− 0.11 (96)	− 0.23* (138)	− 0.25 (96)	− 0.32* (126)	0.42* (66)	− 0.25 (36)	− 0.01 (1032)
PM 2.5	0.39 (42)	0.08 (126)	0.11 (114)	0.38* (60)	0.14 (78)	0.03 (18)	0.35* (72)	0.31* (96)	0.11 (138)	0.16 (96)	− 0.03 (126)	0.01 (66)	0.36 (36)	0.19* (1032)
CVI	0.3 (42)	0.4* (126)	0.57* (114)	0.45* (60)	0.49* (78)	0.51 (18)	0.53* (72)	0.57* (96)	0.43* (138)	0.1 (96)	− 0.24* (126)	0.2 (66)	0.42 (36)	0.3* (1032)
*May*														
Temperature	0.07 (42)	− 0.17 (126)	− 0.1 (114)	− 0.08 (60)	− 0.12 (78)	− 0.51 (18)	− 0.03 (72)	− 0.23 (96)	− 0.35* (138)	0.15 (96)	0.15 (126)	− 0.23 (11)	− 0.07 (36)	− 0.09 (977)
Relative humidity	0.07 (42)	0.14 (126)	− 0.08 (114)	0.28 (60)	− 0.25 (78)	0.09 (18)	− 0.09 (72)	0.13 (96)	0.27* (138)	− 0.01 (96)	− 0.16 (126)	0.56 (11)	0.05 (36)	0.02 (977)
PM 2.5	0.26 (42)	− 0.03 (126)	− 0.05 (114)	0.43* (60)	0.22 (78)	0.29 (18)	0.26 (72)	− 0.18 (96)	− 0.25* (138)	0.47* (96)	− 0.13 (126)	0.64 (11)	0.02 (36)	0.1 (977)
CVI	0.26 (42)	0.11 (126)	0.08 (114)	0.46* (60)	0.35* (78)	0.7* (18)	0.31* (72)	− 0.02 (96)	0 (138)	0.4* (96)	− 0.26* (126)	0.46 (11)	0.18 (36)	0.19* (977)
*June*														
Temperature	0.15 (7)	− 0.09 (126)	0.17 (114)	− 0.04 (60)	− 0.23 (78)	− 0.52 (18)	− 0.33* (72)	0.04 (96)	0.03 (138)	0.05 (96)	0.16 (126)		− 0.21 (6)	0.09 (931)
Relative humidity	0.07 (7)	0.11 (126)	− 0.25* (114)	0.05 (60)	− 0.14 (78)	0.26 (18)	0.08 (72)	− 0.08 (96)	0.04 (138)	0.14 (96)	− 0.14 (126)		0.35 (6)	0.15* (931)
PM 2.5	0.29 (7)	0.24* (126)	0.24 (114)	0.25 (60)	0.27 (78)	− 0.01 (18)	− 0.13 (72)	− 0.05 (96)	0.03 (138)	0.36* (96)	− 0.01 (126)		− 0.36 (6)	0.12 (931)
CVI	0.24 (7)	0.32* (126)	0.15 (114)	0.37* (60)	0.49* (78)	0.46 (18)	0.16 (72)	− 0.07 (96)	− 0.02 (138)	0.26 (96)	− 0.15 (126)		− 0.11 (6)	− 0.01 (931)

^*^Denotes statistically significant correlations at 99% c.l

**Table 2:** Correlation coefficients between the number of new COVID-19 cases during a 5-day period and the mean 5 days temperature, relative humidity, PM2.5 and CVI for the bi-monthly periods March–April, May–June and the 4-month period March–June 2020

	Portugal (7 regions)	Spain (19 regions)	Italy (21 regions)	U.K. (10 regions)	France (13 regions)	Belgium (3 regions)	Netherlands (12 regions)	Germany (15 regions)	Swiss (23 regions)	Poland (15 regions)	Sweden (21 regions)	Norway (11 regions)	Balcans (6 regions)	Europe (176 regions)
March–April														
Temperature	− **0.14 (77)**	− 0.38* (231)	− 0.59* (209)	**0.21 (110)**	**− 0.18 (143)**	**0.21 (33)**	**0.22 (132)**	**− 0.05 (176)**	− 0.26* (253)	0.38* (176)	0.41* (231)	**− 0.19 (121)**	**0.21 (64)**	**− 0.13 (1892)**
Relative humidity	**0.27 (77)**	**− 0.16 (231)**	**− 0.11 (209)**	**− 0.02 (110)**	− 0.56* (143)	− 0.72* (33)	− 0.62* (132)	− 0.43* (176)	**− 0.11 (253)**	− 0.57* (176)	− 0.48* (231)	0.3* (121)	**− 0.25 (64)**	**− 0.06 (1892)**
PM 2.5	**0.27 (77)**	0.19* (231)	0.26* (209)	0.44* (110)	0.28* (143)	0.52* (33)	0.5* (132)	0.31* (176)	0.21* (253)	**− 0.07 (176)**	**− 0.14 (231)**	**− 0.14 (121)**	0.43* (64)	0.21* (1892)
CVI	**0.16 (77)**	**0.13 (231)**	0.3* (209)	0.44* (110)	0.23* (143)	0.51* (33)	0.47* (132)	0.31* (176)	0.21* (253)	**− 0.09 (176)**	− 0.18* (231)	**− 0.03 (121)**	0.47* (64)	0.21* (1892)
May–June														
Temperature	**0.09 (49)**	− 0.22* (252)	**− 0.07 (228)**	**− 0.16 (120)**	− 0.24* (156)	− 0.68* (36)	− 0.36* (144)	− 0.29* (192)	− 0.23* (276)	0.33* (192)	0.32* (252)	**− 0.23 (11)**	**− 0.07 (42)**	**0.11 (1908)**
Relative humidity	**0.06 (49)**	**0.05 (252)**	**− 0.1 (228)**	**0.07 (120)**	− 0.22* (156)	**− 0.21 (36)**	**− 0.11 (144)**	**− 0.02 (192)**	**0.14 (276)**	0.26* (192)	− 0.19* (252)	**0.56 (11)**	**0.05 (42)**	**0.13 (1908)**
PM 2.5	**0.32 (49)**	**0.14 (252)**	**0.1 (228)**	**0.22 (120)**	0.27* (156)	**− 0.11 (36)**	**− 0.06 (144)**	− 0.23* (192)	− 0.16* (276)	0.33* (192)	**0.11 (252)**	**0.65 (11)**	**− 0.02 (42)**	**0.1 (1908)**
CVI	**0.27 (49)**	**0.07 (252)**	**0.07 (228)**	0.25* (120)	0.24* (156)	**− 0.15 (36)**	**− 0.09 (144)**	− 0.24* (192)	**− 0.15 (276)**	0.36* (192)	**0.1 (252)**	**0.64 (11)**	**0.02 (42)**	**0.11 (1908)**
March–June														
Temperature	**0.02 (126)**	− 0.5* (483)	− 0.6* (437)	**− 0.09 (230)**	− 0.43* (299)	− 0.46* (69)	− 0.37* (276)	− 0.42* (368)	− 0.42* (529)	0.35* (368)	0.49* (483)	**− 0.21 (132)**	**− 0.07 (106)**	**0.11 (3800)**
Relative humidity	**0.15 (126)**	**− 0.13 (483)**	**0.13 (437)**	**0.02 (230)**	− 0.39* (299)	− 0.39* (69)	− 0.45* (276)	− 0.38* (368)	− 0.23* (529)	0.27* (368)	− 0.3* (483)	0.3* (132)	**− 0.2 (106)**	**0.09 (3800)**
PM 2.5	0.29* (126)	0.33* (483)	0.29* (437)	0.36* (230)	0.37* (299)	**0.28 (69)**	0.16* (276)	**0.11 (368)**	0.27* (529)	0.15* (368)	**0.04 (483)**	**− 0.13 (132)**	0.4* (106)	**0.05 (3800)**
CVI	**0.2 (126)**	0.26* (483)	0.33* (437)	0.37* (230)	0.35* (299)	**0.29 (69)**	**0.14 (276)**	**0.12 (368)**	0.27* (529)	0.18* (368)	**0.02 (483)**	**− 0.01 (132)**	0.4* (106)	**0.05 (3800)**

^*^Denotes statistically significant correlations at 99% c.l

In both tables, we have calculated an index (COVID-19 Index, hereafter CVI) to describe the interrelationship between the atmospheric variables discussed above and the number of COVID-19 cases. In order to do so, we use a multiple regression analysis in which the dependent variable COVID-19 index (CVI) is the mean daily number of new cases in each group and the independent variables are the temperature (°C), the relative humidity (%), and the daily anomalies of PM2.5 concentrations (in μg m^−3^) as shown in Eq. 1. The CVI index is correlated with the number of new cases in Europe explaining about 9% of their total variance during March and April, 4% during May and 0% during June (Table 1).

1 $$CVI = a_{0} + a_{1} T + a_{2} \left( {{\text{RH}}} \right) + a_{3} {\text{PM}}2.5$$

The multiple regression analysis for Eq. (1) in Europe takes the form:

2 $${\text{CVI}} = 0.302 - 0.041T - 0.004\left( {{\text{RH}}} \right) + 0.059\left( {{\text{PM}}2.5} \right)$$

$$a_{0} = \, 0.302 \pm 0.023, \, a_{1} = 0.041 \pm 0.005; \, a_{2} = 0.004 \pm 0.001{\text{ and }}a_{3} = 0.059 \pm 0.006$$

To summarize, part of the variance of the reported 5-day averaged number of COVID-19 cases in Europe for March and April can be explained by an index based on the atmospheric variables used. More specifically, temperature and humidity are anti-correlated with the number of COVID-19 cases, while PM2.5 is positively correlated. So, the number of new COVID-19 cases shows an increase at lower temperature (cold), lower humidity (dry) and high concentrations of particulates only during March and April. The correlation between particulates and CVI can be expected in view of the fact that smaller particulates have negative effects on respiratory diseases (Dockery and Pope 1994).

However when we repeated the above calculations for May and June, the correlations proved to be insignificant. More specifically the CVI correlation coefficient for the entire 4-month period March–June in Europe drops to 0.05 (Table 2). These results raise a question on whether the spread of COVID-19 disease depends on atmospheric conditions, or not. Based on the contradicting results between the two different periods (the anthropopause and post-anthropopause), the existence of such a relationship cannot be supported and the above analysis remains inconclusive. The spread of the disease is affected primarily by social distancing and the implementation of lockdown measures (e.g., Bherwani et al. 2020). In fact, we have been witnessing an increase in COVID-19 cases during the summer months of 2020 globally.

## A working hypothesis on acceleration of climate change in Siberia and the Arctic and the possible implications for the emergence of pathogens from the permafrost

An additional mechanism connecting climate change with epidemic infections could be that viruses originating from melting glacier or thawing permafrost in the arctic can be transferred both by the wind and the migratory birds toward lower latitudes, eventually enhancing the complexity for infection dynamics. Similar hypotheses on the release of deadly infection vectors from exposed carcasses due to thawing of permafrost in arctic regions have been discussed for anthrax (Revich and Podolnaya 2011; Revich et al. 2012; Hueffer et al. 2020). The viruses could be hidden in the melting ice and thawing of permafrost regions in the Arctic in view of the evolving global warming and its acceleration in this part of the world (IPCC Intergovernmental Panel on Climate Change 2013). Pathogenic viruses and microbes have been found to survive very long periods of time buried in permafrost regions (Tumpey et al. 2005; Biagini et al. 2012; Legendre et al. 2014). For example Legendre et al. 2014 (Legendre et al. 2014) found a still infectious 30,000-year-old virus (named Pithovirus sibericum) in a Siberian permafrost sample. Tumpey et al., 2005 managed to reconstruct the Spanish influenza virus from a victim that was buried in Alaska since 1918 and Biagini et al. 2012 detected a smallpox related virus (variola virus) in mummies buried in Siberian permafrost from the late 17th to early 18th.

**Fig. 3:**
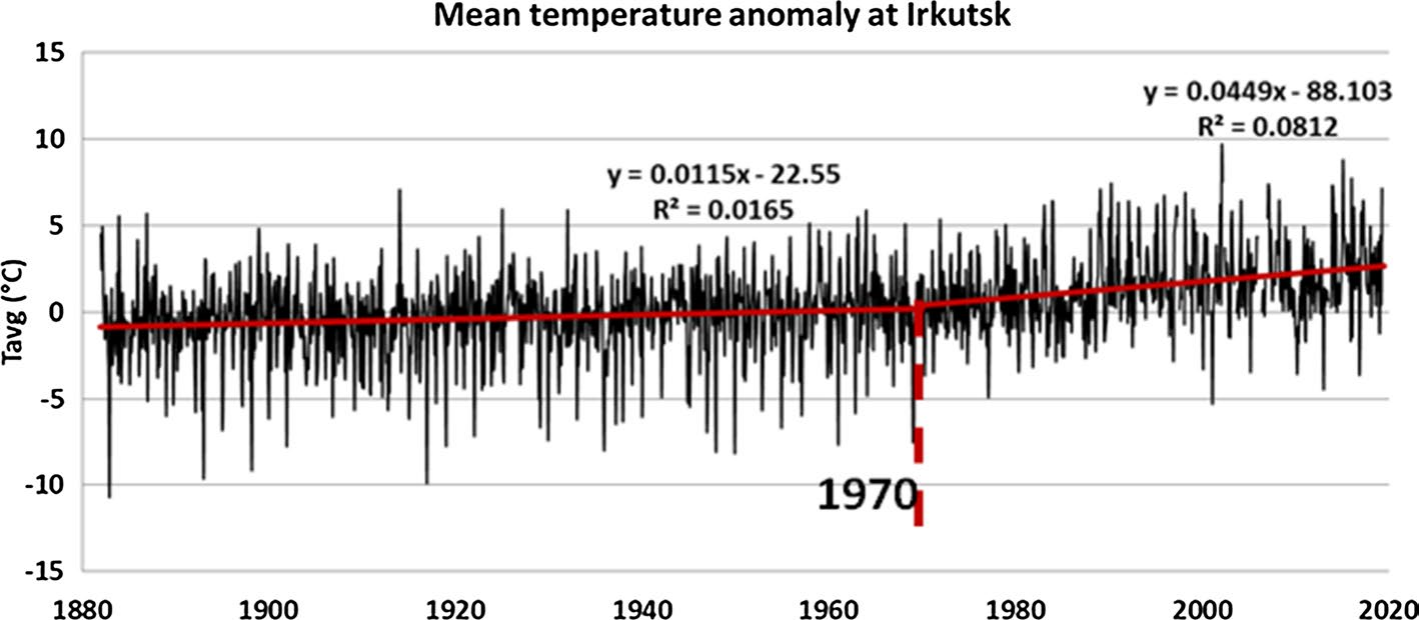
Mean monthly temperature anomalies for Irkutsk (1880–2019)

Permafrost is the soil layer that remains permanently frozen throughout the year under various permafrost types at higher latitudes (i.e., continuous, discontinuous, sporadic and isolated) (Brown et al. 1997). In Central Asia, several mixed types of permafrost start from NW China and Mongolia and extend northward toward Siberia. Long-term measurements of deep permafrost temperatures at depths of 10–200 m in Central Asia and Russia (Romanovsky et al. 2010; Zhao et al. 2010) have shown a continuous warming trend over the last decades (1972–2009). Climate warming leads also in thickening of the active soil layer, i.e., the upper soil region that responds to the seasonal ambient conditions (temperature and precipitation) (Streletskiy et al. 2015). The consecutive melting of permafrost layers from year to year due to climate warming could eventually result to the exposure of gradually deeper permafrost layers and thus increase the possibility for exposure of contaminating sources such as buried carcasses, cemetery graves and fossils along the migrating birds’ pathways and stopover sites (Clairbaux et al. 2019). Lower latitude permafrost areas such as the regions of north Mongolia and south Russia (e.g., Irkutsk, Lake Baikal) are more susceptible to inter-annual temperature changes and to global warming (Hueffer et al. 2020). Following this hypothesis, the acceleration of temperature increase in the Arctic and the melting of permafrost in these areas could be related to the release of present and future “unknown” viruses. That scenario is horrifying, particularly if one considers that migrating bird pathways and wintering areas can also be modified in view of global warming continuation (Romanovsky et al. 2010). From the climatological point of view, Central Asia is one of the most vulnerable regions for manmade climate change. The 140 years (1880–2020) continuous record of mean temperature at the station of Irkutsk (52.27° N, 104.32° E) exhibits a heating of almost 3 °C in this period (Fig. 3). Most of this warming (about 2.5 °C) occurs after 1970 as seen from the right regression line in Fig. 3.

**Fig. 4:**
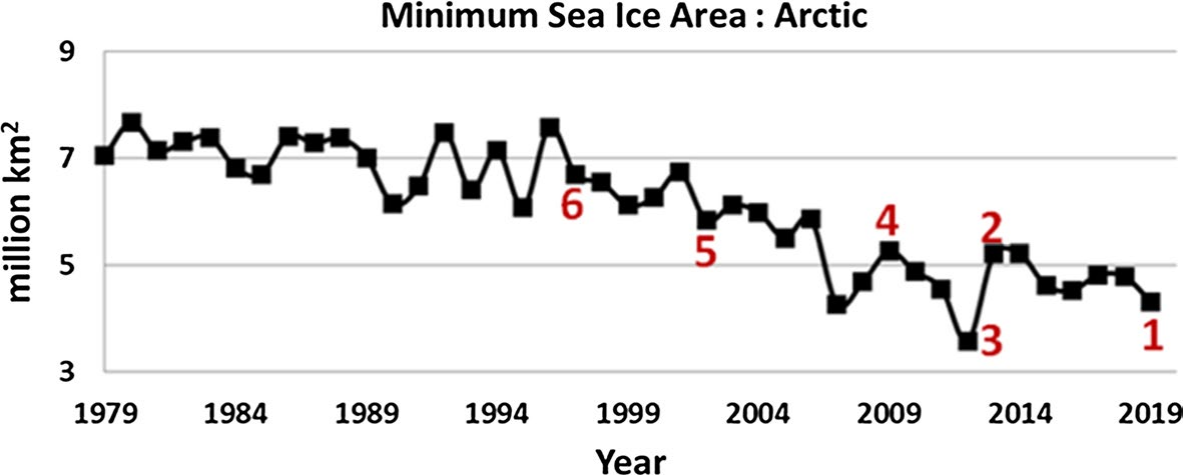
Annual minimum extent of the Arctic Sea Ice (in million km^2^) from 1979 to 2019. Important pandemics during this period are also shown in the graph with red numbers corresponding to Table 3

Warming of the arctic is also clearly evident by the National Snow and Ice Data Center (NSIDC) analysis of satellite observations showing the changes in the arctic sea-ice since 1979 (Fig. 4). The annual minimum of the arctic sea-ice area shrinks from about 7 million km^2^ before 2000 to 4.5 million km^2^ after 2000. The red numbers in Fig. 4 correspond to specific pandemics listed in Table 3. It is worth noticing that as seen in Table 3, seven out of ten major influenza and coronavirus outbreaks in the past 130 years originated in Southeast Asia (Drosten et al. 2003; Ksiazek et al. 2003; Doshi 2011; Zaki et al. 2012; Saunders-Hastings and Krewski 2016; Paraskevis et al. 2020; Wu et al. 2020a, b).

**Table 3:** Historical Pandemics in the northern hemisphere from 1889 to 2019

Pandemic	Onset date	Origin of Outbreak	Type and proposed carrier in the literature (see references)
1. COVID-19	17 November 2019	Wuhan China	Coronavirus, bats, pangolins
2. Bird Flu	19 February 2013	Shanghai China	H7N9, birds, poultry
3. MERS	November 2012	Jeddah Saudi Arabia	Coronavirus, bats, camels
4. Swine Flu	17 April 2009	San Diego, California, USA	H1N1, pigs
5. SARS-CoV	November 2002	Guangdong China	Coronavirus, bats, Civets
6. Bird flu	March 1997	Hong Kong	H5N1, birds, poultry
7. Hong Kong Flu	July 1968	Hong Kong	H3N2, birds, pigs
8. Asian Flu	February 1957	Guizhou China	H2N2, birds
9. Spanish Flu	1918	Possibly China	H1N1, birds, poultry
10. Russian Flu	1889	Asia, Canada and Greenland	H2N2, birds

The continuous decrease in arctic ice cover in the summer months (Fig. 4) will eventually lead to an ice-free polar ocean by the end of this century. This could modify the behavior of several local species including migrating birds. Emerging of new stopover sites previously covered by ice and the ability to prey on the open sea might alter the birds’ habits such as migrating routes and wintering areas (Clairbaux et al. 2019). To emphasize the complexity of this hypothesis, climate warming and consequent changes in permafrost are more evident near lakes and wetlands accompanied also by geological deformations such as landslides along river and lake banks due to permafrost thawing and increase of the active layer depth (Tyszkowski et al. 2015). Such water and wetland ecological complexes are natural resting biotopes for a variety of species like Anseriformes and Gruiformes especially at the south parts of Siberia (Sivay et al. 2012). In a recent study by Sharshov et al. (2017), the virus of influenza was detected in 185 birds from a total of 2300 samples obtained from wild migratory birds in the south of Western Siberia during 2007–2014. Certain viruses including influenza persist in environmental ice (glaciers, snow, permafrost) for years, centuries, millennia, or even longer (Rogers et al. 2004). Therefore, it is possible that a continuous alteration of wetland biotopes landscapes due to climate warming and the consecutive melting of soil layers after centuries of remaining at permafrost stage could assist the re-emergence of ancient virus agents in the environment. The virus agents remain frozen in birds’ feces and are exposed back in spring and summer through melting and thawing processes to generate a seasonal infection cycle (Yu et al. 2010). For the interested reader, we provide in Appendix calculations of back trajectories for the prevailing air masses up to 10 days before the onset of major pandemics. Finally, the extremely rapid urbanization of large regions of biotopes and brutal changes of landscape in the past few decades in China may have also played a role to enhance the genetic diversity of certain pathogens.

## Conclusions

The findings of our study can be summarized as follows:

1. The COVID-19 anthropopause period resulted in a remarkable drop of atmospheric pollutant concentrations in Europe, resembling the anticipated 2050 Green Deal conditions. The comparisons are presented as percentage anomalies from the 2015–2019 average values. The reduction in NO_2_ concentrations is pronounced over the entire continent ranging between − 10 and − 20% in the south and east Europe and − 50% in central Europe. This reduction in NO_2_ is accompanied by an increase of up to 30% in O_3_ as an expected result (titration). The reduction of CO concentrations is also found to be more than − 30% especially in Italy and central Europe with only a few stations presenting a statistical increase (mainly in the Iberian Peninsula). Particulate matter (PM2.5) concentrations also present significant reduction of − 10 to − 20% at most stations.
2. The improvement of air quality in Europe lasted only for 2 months during the lockdown (March and April 2020). As soon as the lockdown measures were waived, the concentrations of pollutants in the atmosphere quickly recovered to pre-pandemic levels. The recovery phase took little more than two months. No climatic implications can be justified from this short-term perturbation of anthropogenic emissions.
3. Our analysis on the possible correlation between meteorology, air quality and the number of daily reported COVID-19 cases in Europe resulted in the construction of a tentative statistical index that explained more than 9% of the total variance in the reported COVID-19 cases for March and April 2020. However, the correlations dropped to insignificant levels when we repeated our calculations for May and June 2020. This shows that any suggested relation between temperature, humidity and virus spread cannot be justified and most likely the global spread of the infection occurs primarily through social contact, traveling, aviation, trade, etc.
4. Seven out of ten influenza and coronavirus pandemics from 1889 to the present originated in China. Significant climate warming, thawing of permafrost soil and glacier retreat is evident in Siberia and the Arctic in coincidence with the increase of epidemics in Southeast Asia. The possible release of frozen viruses at these areas and the possible changes in migrating birds’ routes due to climate change cannot be excluded in studying past and future epidemic hazards.

The significant correlation that was found between virus spread, colder temperatures and lower relative humidity levels during the first two months of the pandemic (March and April 2020) is in accordance with previous correlation studies on the connections between COVID-19 and meteorology for China (Liu et al. 2020a, b; Wang et al. 2020), Iran (Ahmadi et al. 2020), US and Italian regions (Livadiotis 2020) and at global scale (Wu et al. 2020a, b) during the same period. Contradictory results have been reported by a correlation study in Brazil (Auler et al. 2020) where high temperatures and intermediate relative humidity are found to favor the spread of COVID-19. On the other hand, the correlation between the increased PM levels and virus infections is also in agreement with previous COVID-19 studies (Comunian et al. 2020; Fattorini and Regoli 2020; Frontera et al. 2020; Sasidharan et al. 2020). However, as shown in our analysis, such correlations can be misleading because these relations are not robust when repeated for a longer period of time. The persistence of the infection at the north hemisphere during the summer months is also an indication that the variability of COVID-19 cases is not significantly correlated with air temperature variability.

The findings of this paper bring us before new challenges and directions in climate change research. Glacier retreating and thawing of permafrost layers expected in the forthcoming decades may result in new and unknown epidemics posing new horrifying threats to mankind. It is important that combined atmospheric and epidemiological studies as well as detailed scientific expeditions should be organized to investigate such health-related aspects of climate change. Species like bats and other exotic mammals are believed to be the largest reservoir for SARS-CoV-like viruses (Cheng et al. 2007; Cupertino et al. 2020; Fan et al. 2019) and therefore the humanity needs to revisit certain social customs and traditional diets which can threaten our lives and result in pandemic disasters like the one we experience at present.

## Figures and Tables

**Fig. 5:**
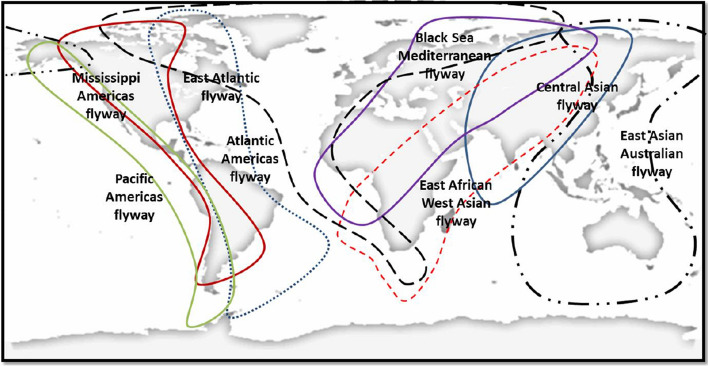
Worldwide distribution of migrating birds pathways (based on Shupeng et al. 2008; Zhang et al. 2014)

**Fig. 6:**
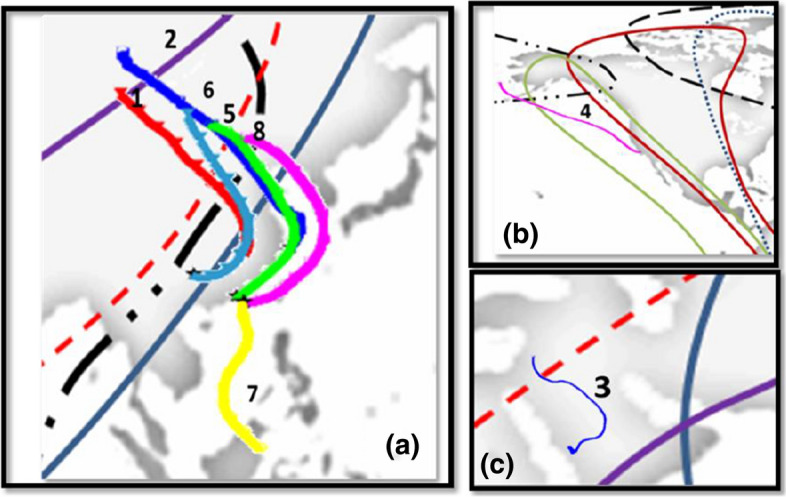
HYSPLIT backtrajectories from origin of epidemic outbreak together with migrating bird pathways (see Fig. 5). The numbers correspond to specific infections in Table 3; **a** Asia; **b** US and Canada **c** Egypt Arabia and Mesopotamia. The starting locations for the backtrajectories are: (1) Wuhan (30.583 N, 114.283E), (2) Shanghai (31.22 N, 121.46E), (3) Jeddah (39.17 N, 21.54E), (4) San-Diego (32.71 N, 117.16 W), (5) Guangdong (23.24 N, 113.30E), (6,7) Hong Kong (22.30 N, 114.18E), (8) Guizhou (26.83 N, 106.83E). No runs were performed for Spanish Flu (1918) and Russian Flu (1889) due to the lack of meteorological data in these eras

## Appendix

See Tables 4 and 5

**Table 4:** Measures taken by each country during COVID-19 pandemic

Country	Measures
Italy	4 March: full closure of all schools and universities nationwide
Spain	14 March: all citizens in quarantine except for those working in healthcare or other vital activities, closing all non-critical businesses
Portugal	March 18: the entirety of the Portuguese territory in a State of Emergency
	April 2–17: extension of the State of Emergency
UK	18 March: Closed schools
	21 March: Closed bars, restaurants, cafes and other entertainment venues
	22 March: Advised vulnerable people to stay at home
	23 March: Initiated Lockdown Phase, Closed most businesses
Germany	16 March: Non-essential public services closed
	22 March: Public gatherings banned
France	13 March: closure of all non-essential public places
	6 March: mandatory home confinement
Belgium	29 January: travel notice advising against non-essential flights to China, Hong Kong excluded
	10 March: the government advised citizens to cancel any indoor scheduled events to be attended by more than 1,000 people for the month of March
	12 March: federal phase of crisis management = closure of schools, discos, cafes and restaurants, cancellation of all public gatherings for sporting, cultural or festive purposes
	17 March: additional measures = Stricter social distancing measures from noon the following day until 5 April, with non-essential travel prohibited, non-essential shops to close, gatherings banned, with penalties for corporate and individual persons who failed to comply with the restrictions
Netherlands	12 March: Gatherings of more than 100 people banned
	13 March: Prison visitations limited to legal affairs
	15 March: All food and beverage outlets, bars, cafes, restaurants, gyms, saunas, sex clubs and coffee shops required to close, except for takeaway and delivery services. Schools closed
	17 March: All education services closed
	23 March: Visits to youth, disability and psychiatric care restricted
	23 March: Ban on non-essential outdoors activities, gatherings with more than 2 people banned, 1.5 m introduced
Poland	12 March—10 April: All schools were closed
	20 March: An official epidemic was declared
	24 March: home restriction
Switzerland	20 March: the government announced that no lockdown would be implemented, but all events or meetings over 5 people were prohibited
	13 March: cancelling of classes in all educational establishments until 4 April 2020, and banning all events (public or private) involving more than 100 people
Sweden	11 March: limiting freedom of assembly by banning all gatherings larger than 500 people
	No mandatory lockdown
Norway	12 March: a national lockdown was announced
	13 March, Norway introduced a ban on visits to Norway through Oslo airport
Greece	10 March: suspension of school operation
	13 March: close down all cafes, bars, museums, shopping centres, sports facilities and restaurants in the country
	6 March, all retail shops were also closed and all services in all areas of religious worship of any religion or dogma were suspended. Supermarkets, pharmacies, food outlets that offer take-away and delivery only, as well as some other businesses, remained open
	23 March: significant restrictions on all nonessential transport and movement
Albania	8 March: all schools for two weeks, ordered cancellation of all large public gatherings, and asked sports federations to cancel scheduled matches
	12 March: 72-h curfew during which only transportation of basic needs such as food and medicine would be permitted, a three-month loan holiday, and the forced closure of garment factories and call centres
	15 March: hardening of its lockdown
Montenegro	13 March: initial round of precautionary measures
Slovenia	16 March: all educational institutions, public transport, all
	restaurants and bars
	20 March: De facto quarantine (with some exemptions)
Croatia	1 February: preparedness measures for the coronavirus epidemic to be prepared for all scenarios
	24 February: additional measures that Croatia will take against the spread of coronavirus, with enhanced control of border crossings to Italy
	3 March: people 60 and those suffering from chronic diseases, according to which they should avoid visiting and entering overcrowded public areas
	16 March: two-week suspension of classes in schools and colleges
Bosnia-Herzegovina	11 March: 2-week shutdown of all schools, high schools and universities
	18 March: an order that banned all public gatherings, suspending the operation of all catering facilities for the preparation and sale of food and beverages, restaurants, pizzerias, confectioneries, beauty salons, hookah bars, coffee bars, discos, tea shops, cafes, private dentists
	20 March: order which banned the movement of people under the age of 18 and over 65
	22 March: a curfew was introduced every day from 18:00 until 05:00

**Table 5:** Pandemic data were obtained (data was retrieved in 27 July 2020) from the following sources:

Country	Data source
Italy	Protezione Civile bulletins at 17:00 CET https://opendatadpc.maps.arcgis.com/apps/opsdashboard/index.html#/b0c68bce2cce478eaac82fe38d4138b1
Spain	Spanish Ministry of Health on confirmed cases of COVID-19 https://cnecovid.isciii.es/covid19/
Portugal	Directorate-General of Health of Portugal (Direção-Geral da Saúde) https://covid19.min-saude.pt/ponto-de-situacao-atual-em-portugal/
U.K	Government of the United Kingdom https://coronavirus.data.gov.uk/
Germany	Robert Koch Institute https://www.rki.de/DE/Content/InfAZ/N/Neuartiges_Coronavirus/Fallzahlen.html
France	Government of France https://dashboard.covid19.data.gouv.fr/vue-d-ensemble?location=FRA
Belgium	Sciensano national public health institute of Belgium https://epistat.wiv-isp.be/covid/
Netherlands	National Institute for Public Health and the Environment
Switzerland	https://www.corona-data.ch/
Sweden	Public Health Agency of Sweden (Folkhälsomyndigheten) https://experience.arcgis.com/experience/09f821667ce64bf7be6f9f87457ed9aa
Norway	Norwegian Institute of Public Health https://www.fhi.no/en/id/infectious-diseases/coronavirus/daily-reports/daily-reports-COVID19/#by-county
Balkans	https://github.com/CSSEGISandData/COVID-19/blob/master/who_covid_19_situation_reports/who_covid_19_sit_rep_time_series/who_covid_19_sit_rep_time_series.csv

### Meteorological conditions before the onset of historical pandemics and a possible hypothesis on pathogens transport by migrating birds

See Figs. 5 and 6

As seen in Fig. 5, the eight identified major migratory routes are mostly meridional and the majority of them extend to more than one continent (Shupeng and van Genderen 2008; Zhang et al. 2014). Wild birds such as waterfowls and cranes follow the Asian flyways in autumn, moving from the northern latitudes of Central and Eastern Asia toward the wintering grounds in China (e.g., Lake Qinghai and Yangtze River delta, see (Wang et al. 2018; Zhang et al. 2014). Particularly in China, a total of 53.6 million hectares of wetlands such as lakes, swamps, rivers as well as artificial wetlands provide breeding and wintering sites for several species including cranes, gulls, ducks, and geese (Wang et al. 2018). A recent study (Deng et al. 2019) shows that the Greater White-fronted Geese autumn migration starts from the Russian Arctic breeding sites in late September moving to wintering areas in southeast China in late October. These worldwide migrating pathways provide a possible natural avian carrier network which should be considered seriously in any mechanism explaining interspecies transmission of viruses. Additionally, earlier studies on the seasonality and origin of pathogenic infections suggest the possibility of long-range airborne transport for influenza virus (Hammond et al. 1989) and transport of avian influenza by dust storms outbreaks (Chen et al. 2010).

In order to investigate the possible atmospheric connections for the historical pandemics of Table 3 we perform a back-trajectory analysis at the onset regions with the Hybrid Single-Particle Lagrangian Integrated Trajectory model (HYSPLIT) (Stein et al. 2015). HYSPLIT runs after 2005 are driven by the Global Data Assimilation System (GDAS) 1° × 1° fields from NOAA’s National Centers for Environmental Prediction Reanalysis data sets (https://ready.arl.noaa.gov/READYcmet.php). The runs before 2005 are driven by NOAA’s CDAS reanalysis dataset (https://psl.noaa.gov/data/gridded/data.ncep.reanalysis.html) that are available at 2.5° × 2.5° grid from 1948 onward. No HYSPLIT runs have been performed for the 1918 Spanish Flu and the 1889 Russian Flu due to the lack of data for these periods. Each backward trajectory is simulated for 10 days starting at 150 m height a.g.l over the infected onset site. The trajectories are calculated at 00:00, 06:00, 12:00 and 18:00 UTC for each day during the month preceding the infection onset. The total of 120 trajectories for each case is filtered to keep only trajectories including points below 100 m a.g.l. This is done in order to exclude the air masses subsiding from upper tropospheric layers without interfering with the boundary layer along their paths. Finally, the trajectories for each case are clustered to a single preferential direction as seen in Fig. 6. The coordinates for the starting point of the trajectories nearest to the reported epidemic outbreak are also given in Fig. 6 caption.

From the cluster analysis shown in Fig. 6, the 5 out of 6 outbreaks which occurred in China were associated with air masses arriving from the northern sector (Mongolia, Siberia). These backtrajectories are seen to intersect the three major migrating birds’ flyways in Asia (Central Asian, East Asian–Australian and East African–West Asian). The onset season of the epidemics reported in Table 3, mainly autumn and winter, coincide with the southward migration of waterfowl species from their breeding summer regions toward their wintering places in China (G. Wang et al. 2008; Wille et al. 2017). The only exception is the Hong Kong flu of 1968 (yellow line in Fig. 6a) that is reported to outbreak in July 1968. For this pandemic Saunders-Hastings and Krewski, 2016 have shown that the responsible H3N2 agent was the result of an antigenic shift (i.e., merging of different strains of a virus to form a new virus subtype) on the H2N2 virus that caused the 1957 Asian flu in the same area which can explain the local outburst of the infection during a summer month. Caution should be given also to the San Diego back trajectory in Fig. 6b (magenta line) which is associated with the outbreak of the Swine Flu pandemic in April 2009. Its direction is again from the north extending up to the Aleutian Islands in the Arctic and interfering with the East Asian–Australian, Pacific Americas and Mississippi Americas flyways. Finally, the MERS 2012 trajectory (blue line in Fig. 6c) indicates a mostly regional origin of the air masses arriving in Jeddah during November 2012 and also possible interference with the Black Sea—Mediterranean flyway. Recirculation of the viruses along the annually migration flyways and spillover of the infection to other avian and mammalian hosts adds to the complexity of epidemic dynamics and the need to clarify its possible association with atmospheric processes. We caution that our trajectory analysis provides only an indication that air mass intersection with the aerial flyways could facilitate virus transport before the onset of the epidemics under study. It is obvious that the actual processes that are responsible for the initiation of an epidemic at a specific place and time are very complex and include a number of biological and social parameters. The possible role of migrating birds needs further elucidation (Tsiodras et al. 2008) and could be considered as additional parameter to this complexity.

## References

[CR1] Ahmadi M, Sharifi A, Dorosti S, Jafarzadeh Ghoushchi S, Ghanbari N (2020). Investigation of effective climatology parameters on COVID-19 outbreak in Iran. Science of The Total Environment.

[CR2] Auler AC, Cássaro FAM, da Silva VO, Pires LF (2020). Evidence that high temperatures and intermediate relative humidity might favor the spread of COVID-19 in tropical climate: A case study for the most affected Brazilian cities. Science of The Total Environment.

[CR3] Benedetti F, Pachetti M, Marini B, Ippodrino R, Gallo RC, Ciccozzi M, Zella D (2020). Inverse correlation between average monthly high temperatures and COVID-19-related death rates in different geographical areas. Journal of Translational Medicine.

[CR4] Bherwani H, Nair M, Musugu K, Gautam S, Gupta A, Kapley A, Kumar R (2020). Valuation of air pollution externalities: comparative assessment of economic damage and emission reduction under COVID-19 lockdown. Air Quality, Atmosphere and Health.

[CR5] Biagini P, Thèves C, Balaresque P, Géraut A, Cannet C, Keyser C (2012). Variola virus in a 300-year-old siberian mummy. New England Journal of Medicine.

[CR6] Briz-Redón Á, Serrano-Aroca Á (2020). A spatio-temporal analysis for exploring the effect of temperature on COVID-19 early evolution in Spain. The Science of the total environment.

[CR7] Brown, J., Ferrians Jr., O. J., & Heginbottom, J. A. (1997). Circum-Arctic Map of permafrost and ground ice conditions: U.S. Geological Survey, Map CP-45, scale *1, *10,000,000. 10.3133/cp45.

[CR8] Chen PS, Tsai FT, Lin CK, Yang CY, Chan CC, Young CY, Lee CH (2010). Ambient influenza and avian influenza virus during dust storm days and background days. Environmental Health Perspectives.

[CR9] Cheng VCC, Lau SKP, Woo PCY, Kwok YY (2007). Severe acute respiratory syndrome coronavirus as an agent of emerging and reemerging infection. Clinical Microbiology Reviews.

[CR10] Clairbaux M, Fort J, Mathewson P, Porter W, Strøm H, Grémillet D (2019). Climate change could overturn bird migration: Transarctic flights and high-latitude residency in a sea ice free Arctic. Scientific Reports.

[CR11] Comunian S, Dongo D, Milani C, Palestini P (2020). Air Pollution and COVID-19: The role of particulate matter in the spread and increase of COVID-19’s morbidity and mortality. International Journal of Environmental Research and Public Health.

[CR12] Cupertino M, Resende M, Mayer N, Carvalho L, Siqueira-Batista R (2020). Emerging and re-emerging human infectious diseases: A systematic review of the role of wild animals with a focus on public health impact. Asian Pacific Journal of Tropical Medicine.

[CR13] Demongeot J, Flet-Berliac Y, Seligmann H (2020). Temperature decreases spread parameters of the new Covid-19 case dynamics. Biology.

[CR14] Deng X, Zhao Q, Fang L, Xu Z, Wang X, He H (2019). Spring migration duration exceeds that of autumn migration in Far East Asian Greater White-fronted Geese (Anser albifrons). Avian Research.

[CR15] Dockery DW, Pope CA (1994). Acute respiratory effects of particulate air pollution. Annual Review of Public Health.

[CR16] Doshi P (2011). The elusive definition of pandemic influenza. Bulletin of the World Health Organization.

[CR17] Drosten C, Günther S, Preiser W, Van der Werf S, Brodt HR, Becker S (2003). Identification of a novel coronavirus in patients with severe acute respiratory syndrome. New England Journal of Medicine.

[CR18] Dutheil F, Baker JS, Navel V (2020). COVID-19 as a factor influencing air pollution?. Environmental Pollution.

[CR19] Fan Y, Zhao K, Shi ZL, Zhou P (2019). Bat coronaviruses in China. Viruses.

[CR20] Fattorini D, Regoli F (2020). Role of the chronic air pollution levels in the Covid-19 outbreak risk in Italy. Environmental Pollution.

[CR21] Finlayson-Pitts BJ, Pitts JN (1997). Tropospheric air pollution: Ozone, airborne toxics, polycyclic aromatic hydrocarbons, and particles. Science.

[CR22] Frontera A, Cianfanelli L, Vlachos K, Landoni G, Cremona G (2020). Severe air pollution links to higher mortality in COVID-19 patients: The “double-hit” hypothesis. Journal of Infection.

[CR23] Gautam.  (2020). The Influence of COVID-19 on Air Quality in India: A Boon or Inutile. Bulletin of Environmental Contamination and Toxicology.

[CR24] Gautam S (2020). COVID-19: Air pollution remains low as people stay at home. Air Quality, Atmosphere and Health.

[CR25] GautamDilwaliya SNK, Srivastava A, Kumar S, Bauddh K, Siingh D (2020). Temporary reduction in air pollution due to anthropogenic activity switch-off during COVID-19 lockdown in northern parts of India. Environment, Development and Sustainability..

[CR26] Gautam S, Hens L (2020). COVID-19: impact by and on the environment, health and economy. Environment, Development and Sustainability.

[CR27] Gunthe SS, Swain B, Patra SS, Amte A (2020). On the global trends and spread of the COVID-19 outbreak: Preliminary assessment of the potential relation between location-specific temperature and UV index. Journal of Public Health.

[CR28] Gupta A, Bherwani H, Gautam S, Anjum S, Musugu K, Kumar N (2020). Air pollution aggravating COVID-19 lethality? Exploration in Asian cities using statistical models. Environment, Development and Sustainability..

[CR29] Hammond GW, Raddatz RL, Gelskey DE (1989). Impact of atmospheric dispersion and transport of viral aerosols on the epidemiology of influenza. Reviews of Infectious Diseases.

[CR30] Hemmes JH, Winkler KC, Kool SM (1960). Virus survival as a seasonal factor in influenza and poliomyelitis. Nature.

[CR31] Hersbach H, Bell B, Berrisford P, Hirahara S, Horányi A, Muñoz-Sabater J (2020). The ERA5 global reanalysis. Quarterly Journal of the Royal Meteorological Society.

[CR32] Huang Z, Huang J, Gu Q, Du P, Liang H, Dong Q (2020). Optimal temperature zone for the dispersal of COVID-19. Science of The Total Environment.

[CR33] Hueffer K, Drown D, Romanovsky V, Hennessy T (2020). Factors contributing to anthrax outbreaks in the circumpolar North. EcoHealth.

[CR34] IPCC Intergovernmental Panel on Climate Change. (2013). Climate Change 2013: The physical science basis. Retrieved from https://www.ipcc.ch/report/ar5/wg1/#.UlAmTj2CjIU.

[CR35] Kissler SM, Tedijanto C, Goldstein E, Grad YH, Lipsitch M (2020). Projecting the transmission dynamics of SARS-CoV-2 through the postpandemic period. Science.

[CR36] Ksiazek TG, Erdman D, Goldsmith CS, Zaki SR, Peret T, Emery S (2003). A novel coronavirus associated with severe acute respiratory syndrome. New England Journal of Medicine.

[CR37] Lal P, Kumar A, Kumar S, Kumari S, Saikia P, Dayanandan A (2020). The dark cloud with a silver lining: Assessing the impact of the SARS COVID-19 pandemic on the global environment. Science of The Total Environment.

[CR38] Legendre M, Bartoli J, Shmakova L, Jeudy S, Labadie K, Adrait A (2014). Thirty-thousand-year-old distant relative of giant icosahedral DNA viruses with a pandoravirus morphology. Proceedings of the National Academy of Sciences of the United States of America.

[CR39] Lin K, Yee-Tak Fong D, Zhu B, Karlberg J (2006). Environmental factors on the SARS epidemic: Air temperature, passage of time and multiplicative effect of hospital infection. Epidemiology and infection.

[CR40] Liu J, Zhou J, Yao J, Zhang X, Li L, Xu X (2020). Impact of meteorological factors on the COVID-19 transmission: A multi-city study in China. Science of The Total Environment.

[CR41] Liu Z, Ciais P, Deng Z, Lei R, Davis SJ, Feng S (2020). Near-real-time monitoring of global CO_2_ emissions reveals the effects of the COVID-19 pandemic. Nature Communications.

[CR42] Livadiotis G (2020). Statistical analysis of the impact of environmental temperature on the exponential growth rate of cases infected by COVID-19. PLoS ONE.

[CR43] Menebo MM (2020). Temperature and precipitation associate with Covid-19 new daily cases: A correlation study between weather and Covid-19 pandemic in Oslo. Norway. Science of The Total Environment.

[CR44] Muhammad S, Long X, Salman M (2020). COVID-19 pandemic and environmental pollution: A blessing in disguise?. Science of The Total Environment.

[CR45] Ogen Y (2020). Assessing nitrogen dioxide (NO2) levels as a contributing factor to coronavirus (COVID-19) fatality. Science of The Total Environment.

[CR46] Paraskevis D, Kostaki EG, Magiorkinis G, Panayiotakopoulos G, Sourvinos G, Tsiodras S (2020). Full-genome evolutionary analysis of the novel corona virus (2019-nCoV) rejects the hypothesis of emergence as a result of a recent recombination event. Infection, Genetics and Evolution.

[CR47] Revich BA, Podolnaya MA (2011). Thawing of permafrost may disturb historic cattle burial grounds in East Siberia. Global Health Action.

[CR48] Revich B, Tokarevich N, Parkinson AJ (2012). Climate change and zoonotic infections in the Russian arctic. International Journal of Circumpolar Health.

[CR49] Rogers SO, Starmer WT, Castello JD (2004). Recycling of pathogenic microbes through survival in ice. Medical hypotheses.

[CR50] Romanovsky VE, Drozdov DS, Oberman NG, Malkova GV, Kholodov AL, Marchenko SS (2010). Thermal state of permafrost in Russia. Permafrost and Periglacial Processes.

[CR51] Şahin M (2020). Impact of weather on COVID-19 pandemic in Turkey. The Science of the total environment.

[CR52] Sasidharan M, Singh A, Torbaghan ME, Parlikad AK (2020). A vulnerability-based approach to human-mobility reduction for countering COVID-19 transmission in London while considering local air quality. Science of The Total Environment.

[CR53] Saunders-Hastings PR, Krewski D (2016). Reviewing the history of pandemic influenza: Understanding patterns of emergence and transmission. Pathogens.

[CR54] Sharshov KA, Yurlov AK, Li X, Wang W, Li L, Bi Y (2017). Avian influenza virus ecology in wild birds of Western Siberia. Avian Research.

[CR55] Shupeng C, van Genderen J (2008). Digital earth in support of global change research. International Journal of Digital Earth.

[CR56] Sivay MV, Sayfutdinova SG, Sharshov KA, Alekseev AY, Yurlov AK, Runstadler J, Shestopalov AM (2012). Surveillance of influenza: A virus in wild birds in the Asian portion of Russia in 2008. Avian diseases.

[CR57] Sobral MFF, Duarte GB, da Penha Sobral AIG, Marinho MLM, de Souza Melo A (2020). Association between climate variables and global transmission oF SARS-CoV-2. The Science of the total environment.

[CR58] Stein AF, Draxler RR, Rolph GD, Stunder BJB, Cohen MD, Ngan F (2015). Noaa’s hysplit atmospheric transport and dispersion modeling system. Bulletin of the American Meteorological Society.

[CR59] Streletskiy D, Anisimov O, Vasiliev A (2015). Permafrost Degradation. Snow and Ice-Related Hazards, Risks, and Disasters.

[CR60] Tsiodras S, Kelesidis T, Kelesidis I, Bauchinger U, Falagas ME (2008). Human infections associated with wild birds. Journal of Infection.

[CR61] Tumpey TM, Basler CF, Aguilar PV, Zeng H, Solórzano A, Swayne DE (2005). Characterization of the reconstructed 1918 Spanish influenza pandemic virus. Science.

[CR62] Tyszkowski S, Kaczmarek H, Słowiński M, Kozyreva E, Brykała D, Rybchenko A, Babicheva VA (2015). Geology, permafrost, and lake level changes as factors initiating landslides on Olkhon Island (Lake Baikal, Siberia). Landslides.

[CR63] Wang G, Zhan D, Li L, Lei F, Liu B, Liu D (2008). H5N1 avian influenza re-emergence of Lake Qinghai: Phylogenetic and antigenic analyses of the newly isolated viruses and roles of migratory birds in virus circulation. Journal of General Virology.

[CR64] Wang Chen, Horby PW, Hayden FG, George FG (2020). A novel coronavirus outbreak of global health concern. Lancet.

[CR65] Wang J, Tang K, Feng K, Lv W (2020). High temperature and high humidity reduce the transmission of COVID-19. SSRN Electronic Journal.

[CR66] Wang X, Cao L, Bysykatova I, Xu Z, Rozenfeld S, Jeong W (2018). The Far East taiga forest: Unrecognized inhospitable terrain for migrating Arctic-nesting waterbirds?. PeerJ.

[CR67] Wille M, Latorre-Margalef N, Waldenström J (2017). Of Ducks and men: Ecology and evolution of a zoonotic pathogen in a wild reservoir host. Modeling the Transmission and Prevention of Infectious Disease.

[CR68] World Health Organization (WHO) (2020). Coronavirus disease 2019 Situation Report 51 11th March 2020. World Health Organization.

[CR69] Wu F, Zhao S, Yu B, Chen YM, Wang W, Song ZG (2020). A new coronavirus associated with human respiratory disease in China. Nature.

[CR70] Wu Y, Jing W, Liu J, Ma Q, Yuan J, Wang Y (2020). Effects of temperature and humidity on the daily new cases and new deaths of COVID-19 in 166 countries. Science of The Total Environment.

[CR71] Marchenko VY, Alekseev AY, Tserennorov D, Yurlov AK, Susloparov IM, Sharshov KA (2010). Results of the influenza virus surveillance in wild birds in Western part of Mongolia. Asian Pacific Journal of Tropical Medicine.

[CR72] Zaki AM, Van Boheemen S, Bestebroer TM, Osterhaus ADME, Fouchier RAM (2012). Isolation of a novel coronavirus from a man with pneumonia in Saudi Arabia. New England Journal of Medicine.

[CR73] Zhang J, Jin Z, Sun GQ, Sun XD, Wang YM, Huang B (2014). Determination of original infection source of H7N9 avian influenza by dynamical model. Scientific Reports.

[CR74] Zhao L, Wu Q, Marchenko SS, Sharkhuu N (2010). Thermal state of permafrost and active layer in central Asia during the international polar year. Permafrost and Periglacial Processes.

[CR75] Zoran MA, Savastru RS, Savastru DM, Tautan MN (2020). Assessing the relationship between surface levels of PM2.5 and PM10 particulate matter impact on COVID-19 in Milan, Italy. The Science of the total environment.

[CR76] Zoran MA, Savastru RS, Savastru DM, Tautan MN (2020). Assessing the relationship between ground levels of ozone (O_3_) and nitrogen dioxide (NO_2_) with coronavirus (COVID-19) in Milan, Italy. The Science of the total environment.

